# Electrospun Scaffolds in Periodontal Wound Healing

**DOI:** 10.3390/polym13020307

**Published:** 2021-01-19

**Authors:** Mária Budai-Szűcs, Marco Ruggeri, Angela Faccendini, Attila Léber, Silvia Rossi, Gábor Varga, Maria Cristina Bonferoni, Péter Vályi, Katalin Burián, Erzsébet Csányi, Giuseppina Sandri, Franca Ferrari

**Affiliations:** 1 Institute of Pharmaceutical Technology and Regulatory Affairs, Faculty of Pharmacy, University of Szeged, Eötvös u. 6, H-6720 Szeged, Hungary; budai-szucs.maria@szte.hu (M.B.-S.); leber.attila@gmail.com (A.L.); soosne.csanyi.erzsebet@szte.hu (E.C.); 2 Department of Drug Sciences, University of Pavia, Viale Taramelli 12, 27100 Pavia, Italy; marco.ruggeri02@universitadipavia.it (M.R.); angela.faccendini@gmail.com (A.F.); silvia.rossi@unipv.it (S.R.); cbonferoni@unipv.it (M.C.B.); franca.ferrari@unipv.it (F.F.); 3 Department of Organic Chemistry, University of Szeged, Dóm tér 8, H-6720 Szeged, Hungary; gabor.varga5@chem.u-szeged.hu; 4 Department of Periodontology, Faculty of Dentistry, University of Szeged, H-6720 Szeged, Hungary; valyi.peter@stoma.u-szeged.hu; 5 Institute of Clinical Microbiology, Faculty of Medicine, University of Szeged, H-6720 Szeged, Hungary; burian.katalin@med.u-szeged.hu

**Keywords:** periodontitis, gelatin, chitosan, alginate, nanofibrous scaffold, wound healing, antibacterial properties

## Abstract

Periodontitis is a set of inflammatory conditions affecting the tissues surrounding the teeth predominantly sustained by bacterial infections. The aim of the work was the design and the development of scaffolds based on biopolymers to be inserted in the periodontal pocket to restore tissue integrity and to treat bacterial infections. Nanofibrous scaffolds were prepared by means of electrospinning. Gelatin was considered as base component and was associated to low and high molecular weight chitosans and alginate. The scaffolds were characterized by chemico–physical properties (morphology, solid state-FTIR and differential scanning calorimetry (DSC)-surface zeta potential and contact angle), and mechanical properties. Moreover, preclinical properties (cytocompatibility, fibroblast and osteoblast adhesion and proliferation and antimicrobial properties) were assessed. All the scaffolds were based on cylindrical and smooth nanofibers and preserved their nanofibrous structure upon hydration independently of their composition. They possessed a high degree of hydrophilicity and negative zeta potentials in a physiological environment, suitable surface properties to enhance cell adhesion and proliferation and to inhibit bacteria attachment. The scaffold based on gelatin and low molecular weight chitosan proved to be effective in vitro to support both fibroblasts and osteoblasts adhesion and proliferation and to impair the proliferation of *Streptococcus mutans* and *Aggregatibacter actinomycetemcomitans*, both pathogens involved in periodontitis.

## 1. Introduction

Periodontitis is a set of inflammatory conditions affecting the tissues surrounding the teeth and is predominantly a bacterial infection that affects the protective and supportive tissues of the tooth. This is a great health problem and nearly half of the adult population are affected. Periodontitis can reduce patients’ quality of life and cause tooth loss, disability, masticatory dysfunction as well. It can be also associated with systemic chronic inflammatory diseases, such as atherogenic cardiovascular disease, rheumatoid arthritis, chronic kidney disease, type 2 diabetes mellitus, and chronic obstructive pulmonary disease. As a complex disease, its onset depends on etiological and modifiable risk factors e.g., smoking, poor oral hygiene, poorly controlled diabetes mellitus, obesity, and stress [1,2,3].

In periodontitis, bacteria accumulate on the non-shedding surfaces of the oral cavity, which initiate and trigger a dysfunctional inflammatory immune response destroying the underlying supporting tissues. The first stage is gingivitis, where the bacteria cause inflammation with sensitive, red, and bleeding gums. Unless gingivitis is treated, the inflammation progresses to the second stage which is periodontitis. During the progression of the disease the destruction of the gingival tissues, the resorption of the alveolar bone, the migration of the junctional epithelium toward the roots, and the formation of periodontal pocket occur. If the inflammation exists permanently, the progression of the disease results in the deepening of the periodontal pocket and finally tooth loss.

The possible treatment of periodontitis involves various non-surgical and surgical methods in order to reduce periodontal pocket depth, eliminate residual plaque, and to initiate the regeneration of periodontal supporting tissues. The non-surgical methods include mechanical debridement and antimicrobial pharmacotherapy. The mechanical debridement can be effective, but it is not adequate for removing all microorganisms, especially in the subgingival area, and this can lead to reinfection and disease progression [4,5]. The effectiveness of the mechanical therapy can be improved using local antimicrobials [6]. However, in the case of local pharmacotherapy, the risks related to a microbial resistance are high since it is difficult to effectively reach the site of action at a constant and effective concentration for a sufficient duration [7].

Nanotechnology-based nanobiomaterials have recently been recognized as a novel approach for treatment of periodontal disease due to the capability of supporting tissue regeneration often associated with antimicrobial properties [8]. Liposomes [9], polymeric nanoparticles (i.e., chitosan-based), inorganic nanoparticles/nanocrystals (i.e., Ag nanoparticles, hydroxyapatite-based) [8,9], dendrimers (i.e., polyamidoamine-based) [8,9,10] and nanofibers [8,9] have been recently proposed in literature. All these seem to have great potential to improve the efficacy of periodontal disease treatment. In particular electrospun scaffolds are good candidates for the local treatment of periodontitis, thanks to their capability to enhance tissue repair [11], easy production, and cost-effectiveness [12,13,14].

In the literature, there are many examples of antimicrobial drugs such as metronidazole, amoxicillin, ampicillin, tetracycline, doxycycline, and tinidazole loaded in nanofibers based on biodegradable polymers such as polycaprolactone [15,16], poly(l-lactic acid)/poly(d-lactic acid) [17], polylactide [18,19], ε-caprolactone [20,21] and poly(lactic-co-glycolic acid) (PLGA) [22,23] alone or combinations.

An alternative strategy to the local delivery of antimicrobial drugs is biopolymers characterized by bioactive properties to combine wound healing properties with antibacterial ones.

The aim of this work was the design and development of electrospun nanofibrous scaffolds as implantable medical devices, intended for wound healing of the periodontal pocket. Different biopolymers were considered. Gelatin (G), a derivative of collagen, was selected since it preserves the capability to sustain cell adhesion and proliferation typical of collagen without immunogenicity [24], while alginate (A) and chitosan (C), both characterized by antibacterial properties were associated to G to confer antimicrobial properties to the scaffolds and to have a synergic effect on wound healing [25]. The scaffolds were characterized by morphology (scanning electron microscopy (SEM)) and solid-state (infrared spectroscopy (FTIR), X-ray diffraction (XRD), differential scanning calorimetry (DSC), surface zeta potential and contact angle). Furthermore, scaffold biocompatibility was assessed on fibroblasts and osteoblasts (SAOS-2 cells), while scaffold antimicrobial effectiveness was assessed against *Streptococcus mutans* and *Aggregatibacter actinomycetemcomitans*.

## 2. Materials and Methods

### 2.1. Materials

Gelatin (G), from bovine skin, gel strength ~225 g Bloom, Type B, molecule weight ≅ 50 kDa (Sigma Aldrich, Milan, Italy); low molecular weight chitosan (CL) (deacetylated chitin, poly(D-glucosamine)), molecular weight 50-190 kDa (Sigma Aldrich, Milan, Italy), deacetylation degree 76%; high molecular weight chitosan (CH) (β-(1-4)-linked d-glucosamine and N-acetyl-d-glucosamine) deacetylation degree 98%, molecular weight 251 kDa (ChitoClear, Siglufjörður, Iceland); alginic acid sodium salt from brown algae (A), medium viscosity average molecular mass 80−120 kDa, 61% mannuronic residues and 39% guluronic residues, mannuronic/guluronic ratio of 1.56 (Sigma Aldrich, Milan, Italy) were used for scaffold preparation. Citric acid (monohydrated citric acid, EP grade, Carlo Erba, Milan, Italy) was used as crosslinking agent.

### 2.2. Methods

#### 2.2.1. Preparation and Characterization of the Polymeric Blends

The quali-quantitative composition of the polymeric blends is reported in Table 1. For the G scaffold, 20% w/w gelatin solution was prepared in 90:10 acetate buffer (0.1 M, pH 5):acetic acid volume ratio at 40 °C, and, after complete hydration, 5% *w*/*w* citric acid was added to the blend.

For GCL (gelatin-low molecular weight chitosan), GCH (gelatin-high molecular weight chitosan) and GA (gelatin- alginic acid sodium salt) scaffolds, 40% *w*/*w* gelatin was hydrated in water at 40 °C. Separately, 4% *w*/*w* CL in 90:10 acetate buffer (0.1 M, pH 5):acetic acid volume ratio, or 4% *w*/*w* CH in 80:20 water:acetic acid volume ratio, or 4% *w*/*w* A in 80:20 water:acetic acid volume ratio were prepared and 10% *w*/*w* citric acid was added to each solution. Finally, CH or CL or A solutions were blended to a 1:1 weight ratio at 40 °C to prepare GCH, GCL or GA scaffolds.

Conductivity, pH, surface tension and viscosity of each polymeric blend were characterized.

The conductivity was measured using a conductometer (FiveGo FG3 Portable conductometer, Mettler Toledo, Milan, Italy).

The pH was measured with a pH-meter (Mettler Toledo, Milan, Italy).

The surface tension of the polymeric solutions was assessed using a tensiometer (DY-300–Kyowa, Saitama, Japan) with a 0–100 mN/m measurement range at 40 °C by a time-based detection.

The rheological analysis was carried out by means of a rotational rheometer (MCR 102 rheometer, Anton Paar, Turin, Italy) at 40 °C, using a cone plate combination (CP50-1: 50.0 mm diameter and 1° angle) as the measuring system, after 1 min of rest time.

#### 2.2.2. Preparation of Electrospun Scaffolds

The polymer blends G, GCH, GCL, GA, were electrospun using an electrospinning apparatus (STKIT-40, Linari Engineering, Pisa, Italy), equipped with a high voltage generator (3–40 kV), a-10cc glass syringe with a stainless-steel needle, a volumetric pump (Razel R99-E, Linari Engineering, Pisa, Italy) and a planar collector covered with an aluminum foil. The following parameters were used in the electrospinning process: DC (direct current) (voltage) = 28 kV, needle-to-collector distance = 15 cm, flow = 0.794 mL/h, temperature = 40 °C, relative humidity = 10%. The electrospinning time was 1 h and the thickness of the scaffolds ranged from 1.5 to 2.5 mm (caliper resolution 0.01 mm). The crosslinking process was performed by heating at 150 °C for 1 h, to activate citric acid [26,27,28,29,30,31].

#### 2.2.3. Scaffold Characterization

##### Morphology

Scaffold morphology was investigated by means of SEM (Tescan, Mira3XMU, CISRIC, University of Pavia, Brno, Czech Republic) after graphite sputtering. Scaffolds were analyzed after the crosslinking process and after 20 days of hydration and then drying. Nanofiber diameters were determined by image analysis software (ImageJ, ICY, Institute Pasteur, Paris, France).

##### Hydrophilicity

The hydrohilicity of the scaffolds was evaluated by measuring the water contact angles (Contact Angle Meter DMe-211 Plus—Kyowa Interface Science Co, Ltd., Niiza, Japan). Water contact angles were determined using the sessile drop method. An amount of 10 µL of water was dropped on the surface of the scaffold and images of drop onto the scaffold were automatically captured after 1000 ms and the contact angle measured by means of the FAMAS software.

##### FTIR Spectra

Transmission FTIR spectra of each scaffold were recorded with an Avatar 330 FT-IR spectrometer (Thermo Nicolet, Waltham, MA, USA) between 1600 and 600 1/cm at an optical resolution of 16 1/cm. A total of 128 scans were obtained for each sample.

##### Differential Scanning Calorimetry

DSC measurements were carried out with a Mettler-Toledo DSC 821e instrument in argon atmosphere (100 mL/min) in +25–240 °C temperature range using 10 °C/min heating rate. An amount of 1 mg of each sample was put in 40 µL aluminum pans. The tops were holed, then the pans were sealed.

##### Surface Zeta Potential

The apparent zeta potential (ζ) of each scaffold was determined from the measurement of the streaming potential. Streaming potential measurements were performed with SurPASS™ 3 (Anton Paar, Turin, Italy) using a cylindrical cell. The scaffolds (10 × 10 mm^2^) were mounted between two filter disks in the sample holder of the cylindrical cell. KCl aqueous solution of 0.1 mol/L concentration was used as the streaming solvent and its pH was scanned in the range 2–9, to determine the isoelectric point (iep) and the ζ at physiological pH.

##### Mechanical Properties

Mechanical properties of the scaffolds were investigated by means of a TA.XT plus dynamometer (Stable Microsystems, ENCO, Godalming, United Kingdom), equipped with a 1 kg load cell and A/TG tensile grips. Scaffolds (3 cm × 1 cm = 3 cm^2^) were mounted between two grips, the lower one fixed and the upper one movable at a constant rate of 0.5 mm/s. Dry or hydrated scaffolds were stretched up to break and the force was recorded as a function of the movable grip displacement. Moreover, the Elongation (%) and the Young’s modulus (mN/cm^2^) were calculated from the stress vs. strain curves. The mechanical parameters were normalized on scaffold thickness.

##### Biopharmaceutical Characterizations

Cytotoxicity and adhesion assays were carried out using two different cell lines: fibroblasts (normal human dermal fibroblasts, NHDF, from juvenile foreskin, PromoCell, Milan, Italy) and SAOS-2 cells (human primary osteogenic sarcoma cells, Sigma Aldrich, Milan, Italy). Scaffolds were cut to cover the bottom of a well in a 96 well-plate and cells, either fibroblasts or osteoblasts), were seeded onto each scaffold at 10^5^ cells/cm^2^ seeding density. After 6 days of growth, cytocompatibility of scaffolds was evaluated by means of MTT (tetrazolium salt, [3-(4,5-dimethylthiazol-2-yl)-2,5-diphenyltetrazolium bromide]) assay. Briefly, MTT was solubilized at 2.5 mg/mL in phosphate buffer solution (PBS, Sigma-Aldrich, Milan, Italy). At prefixed days, the medium in each well was removed and 50 μL of MTT solution plus 100 μL of PBS were added and subsequently put in contact with the cell substrates at 37 °C for 3 h in the incubator. Then, MTT solution was removed from each well and 100 μL of dimethylsulfoxide (DMSO, Sigma-Aldrich, Milan, Italy) was added. The absorbance was read using an ELISA Plate Reader at λ = 570 nm (with reference λ = 690 nm). Fibroblasts or SAOS-2 (seeding density 10^5^ cells/cm^2^) grown in standard conditions and subjected to the same protocols were used as control (GM, growth medium). Furthermore, the cells adhered to the scaffolds were washed three times with PBS. Then the cell actin cytoskeleton was stained with phalloidin FITC (fluorescein isothiocyanate) Atto 488 (50 μL at 20 μg/mL in PBS in each well, contact time 30 min) (Sigma-Aldrich, Milan, Italy). Subsequently, after three PBS washes, the cell nuclei were stained with Hoechst 33,258 (100 μL of solution at 1:10,000 dilution in PBS per each well, contact time 10 min in the dark) (Sigma-Aldrich, Milan, Italy), for 10 min. After three further PBS washes, the scaffolds were mounted on glass slides, covered using coverslips and analyzed using CLSM (confocal laser scanning microscopy, Leica TCS SP8 DLS, Leica Microsystems, Milan, Italy) at λ_ex_ = 346 nm and λ_em_ = 460 nm for Hoechst 33,258 and λ_ex_ = 501 nm and λ_em_ = 523 nm for phalloidin FITC. The acquired images were processed by means of Leica software (Leica Microsystem, Milan, Italy).

##### Microbiological Investigation

The antimicrobial activity was assessed using two bacterial strains *Streptococcus mutans* (ATCC 25175™) (Gram +) and *Aggregatibacter actinomycetemcomitans* (ATCC 29524™) (Gram −). A bacterial suspension of one McFarland standard concentration, equivalent to approximately 3 × 10^8^ colony forming units CFU/mL in suspension, was freshly prepared in 0.9% NaCl solution. Equivalent portions of the suspensions were spread onto horse blood agar plates, where the circular portions of each scaffold were then placed on. After 24 h of incubation in anaerobic conditions, the diameter of the inhibition zones was measured. Disks were obtained by compressing approximately 15 mg of each scaffold fiber under 1 kN pressure for 30 s in a 13-mm diameter pellet die. This allowed comparison of the antimicrobial effect using the same conditions. The diameter of the inhibition zone was calculated by subtracting the diameter of the disk from the total inhibition zone diameter. A dental gel (CHX gel) from the market which contains 1% chlorhexidine digluconate was applied in the same arrangement (same dose and diameter) as a reference.

#### 2.2.4. Statistical Analysis

Statistical analysis was performed using the Astatsa online statistical calculator. One-way analysis of variance (ANOVA) was followed by the Scheffè test for post-hoc comparisons. *p* < 0.05 was considered significative.

## 3. Results and Discussion

### 3.1. Characterization of the Polymeric Blends

Table 2 reports the conductivity, pH, surface tension, and viscosity values of the polymeric blends.

In the polymeric mixtures the conductivity was about 3700–3900 µS/cm for the G and GCH blends, while it was greater and around 5000 µS/cm in the blends based on low molecular weight chitosan or sodium alginate. However, the *p*-value corresponding to the one-way ANOVA is higher than 0.05, suggesting that the treatments were not significantly different. The polymeric blends, regardless of their composition, had an acidic behavior with pH values ranging from 2.9 to 3.6, and surface tension values were almost similar for all blends. At high shear rates, the addition of chitosan or sodium alginate increased the viscosity of the solution compared to G solution.

### 3.2. Chemico–Physical Characterization

**Figure 1:**
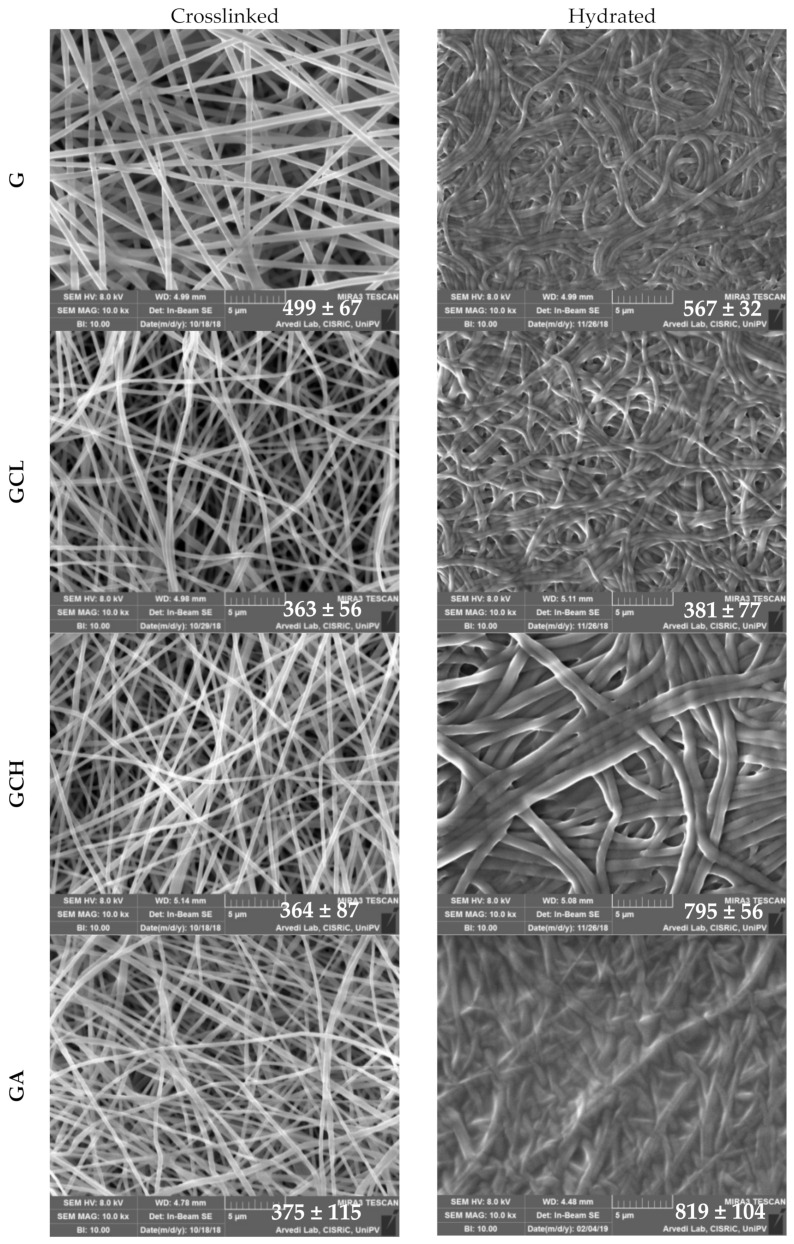
SEM microphotographs of scaffolds after cross-linking and after 20 days hydration in phosphate buffer solution (PBS). In each image, the diameters (nm) are reported (mean values ± sd; *n* = 100). ANOVA one-way; Scheffé test (*p* < 0.05): crosslinked = gelatin (G) vs. GCL, GCH, GA; hydrated: GCL vs. GCH, GA. GCH: dry vs. hydrated; GA: dry vs. hydrated.

Figure 1 reports SEM microphotographs of scaffolds after the heating process and after 20 days of hydration. In each image the nanofiber mean diameters (nm) are reported.

The morphological analysis evidenced a nanofibrous structure for all the scaffolds. In the dry state, scaffolds based on gelatin and polysaccharide blends, GCL, GCH and GA, were made of thin (around 380 nm diameters), cylindrical and uniform nanofibers with a smooth surface. On the contrary the scaffold based on pristine gelatin showed significantly thicker nanofibers (around 500 nm), but they were cylindrical, uniform with a smooth surface anyway. The nanofibrous structure was preserved also upon hydration. However, the presence of CH, in the GCH scaffold, and of A, in the GA scaffold, caused a significant swelling of the nanofibers with an approximately doubling of dimensions (around 800 nm diameters) after hydration. On the contrary, G and GCL scaffolds did not substantially swell and remained almost unchanged in size.

The different behavior could be due to the interaction between gelatin and citric acid in presence of the two chitosan grades or alginate. In particular, if gelatin was not associated to another polymer it is conceivable that there was a strong interaction between the negatively charged carboxylic group of citric acid and the positively charged amino groups of gelatin in G scaffolds.

On the contrary, the presence of alginate seems to interfere with the interaction between gelatin and citric acid since the carboxylic groups of alginate could compete with those of the citric acid, weakening the interpolymer network and allowing a greater degree of polymer chain flexibility, thus swelling the structure in the presence of aqueous medium. The presence of chitosan was characterized by two opposite behaviors: CH having a high deacetylation degree assisted the polymer network consolidation in the nanofibers by increasing the interaction points between negative the carboxylic group of citric acid and amino groups bared by both gelatin and chitosan. Conversely, the low deacetylation degree of CL decreased the interaction points compared to those of CH and simultaneously distanced gelatin from citric acid due to its steric hindrance. The limited swelling of the scaffold is an attractive feature and should favor the cell adhesion, giving a stable support and should avoid the surrounding tissue compression.

**Figure 2:**
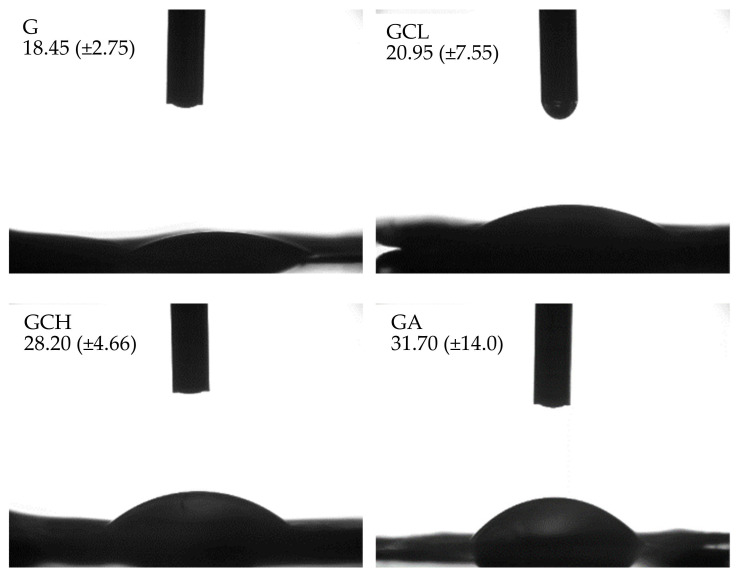
Images of the drop onto the scaffold surface after 1000 ms. In each image the value of the contact angle is reported (mean values ± s.d.; *n* = 3).

Moreover, all the scaffolds were characterized by contact angles lower than 30°, indicating high hydrophilicity (Figure 2). In particular, the presence of polyelectrolyte (either chitosan or alginate) caused an increase in contact angle, although this was not significative. This could be due to a decrease in the hydrophilic moieties of the polysaccharides increasing scaffold hydrophobicity. The deacetylation degree of chitosan seems to contribute to the hydrophilicity/hydrophobicity of the scaffolds: the higher the deacetylation degree, the lower the interaction with gelatin and consequently the higher the hydrophilicity. These results are in agreement with the scaffold morphology upon hydration. The scaffold hydrophilicity should allow cell–scaffold interaction to enhance the effectiveness in tissue healing.

**Figure 3:**
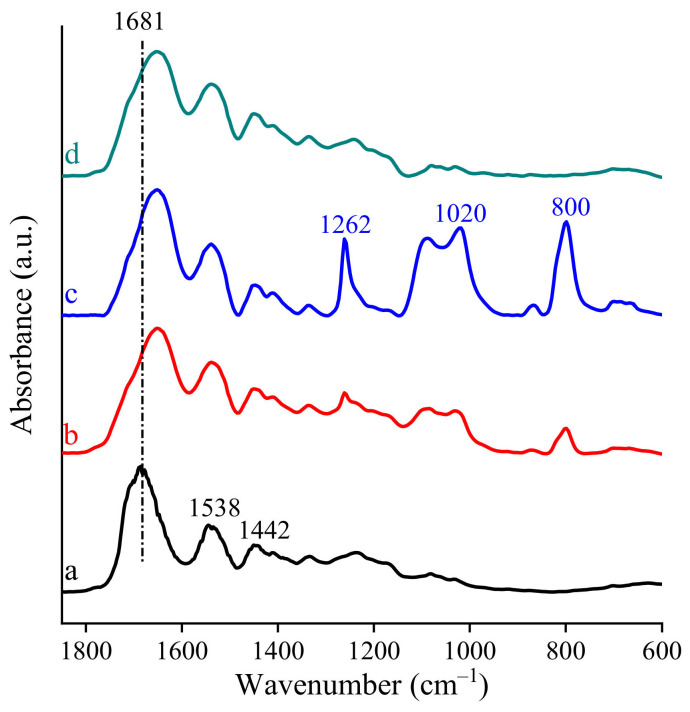
FTIR of nanofibrous scaffolds: (**a**) G; (**b**) GCL; (**c**) GCH and (**d**) GA.

FT-IR analysis suggests that chemical interactions among the different components of the scaffolds occurred (Figure 3).

As reported in the literature, pristine gelatin is characterized by distinctive absorption bands and in particular amide I (~1681 cm^−1^) and amide II (~1538 cm^−1^) as well as a broad peak at about 1442 cm^−1^ associated with symmetrical stretching bond vibration of the carbonyl group overlapped with the deformation mode vibration of the −CH group [32]. In the FT-IR spectra of all the scaffolds, the absorption bands at approximately 1642 and 1541 cm^−1^, which are related to the shifted amide I and amide II vibrations, were attributed to the presence of gelatin. The observed shifts of these characteristic bands indicate that in the scaffolds the interaction among gelatin and the other components was not a simple physical interaction. According to the shape of the broadened amide I vibration, it is conceivable that this peak was overlapped with symmetrical stretching vibration of carboxylate groups of the other components (citric acid or alginate, where it was present). This seems attributable to a chemical interaction among components.

Furthermore, in the spectra of chitosan-based scaffolds, the appearance of the intense peak identified as shifted bending vibration of −OH group at about 1262 cm^−1^ suggests that chitosan formed a strong chemical interaction with gelatin and/or citric acid [33].

**Figure 4:**
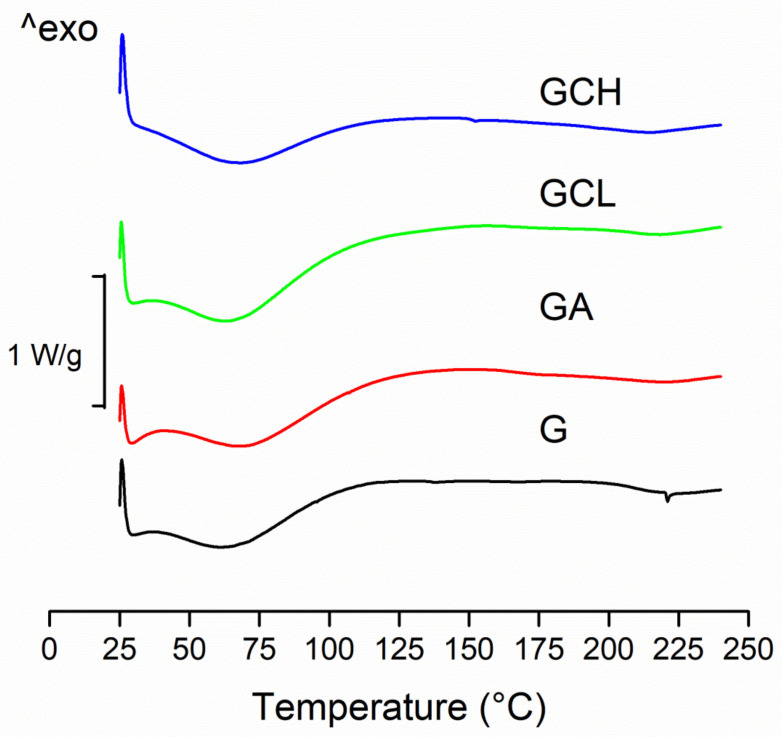
Differential scanning calorimetry DSC thermograms of scaffolds: G, GCL, GCH and GA.

All the scaffolds were characterized by rather similar DSC profiles (Figure 4).

These showed two endothermic peaks. The first broad peak (30–130 °C) corresponds to the water evaporation from the scaffold, while the second endothermic peak corresponds to the melting of the polymer. As fibrous protein, gelatin is plasticized by water: this seems to lower glass transition temperature (*T*_g_) and broadening melting temperature range (*T*_m_). However, the evaporation of residual acetic acid (a volatile solvent) (118 °C boiling temperature) partially concealed the *T*_g_ peak. These observations are argued considering the thermal analysis of pure gelatin, [34], which presents both *T*_g_ and (*T*_m_), and a relatively large endothermic transition (*T*_i_) upon the heating [35].

The presence of chitosans (independently of molecular weight and deacetylation degree) or alginate did not alter the thermal behavior of the gelatin.

**Figure 5:**
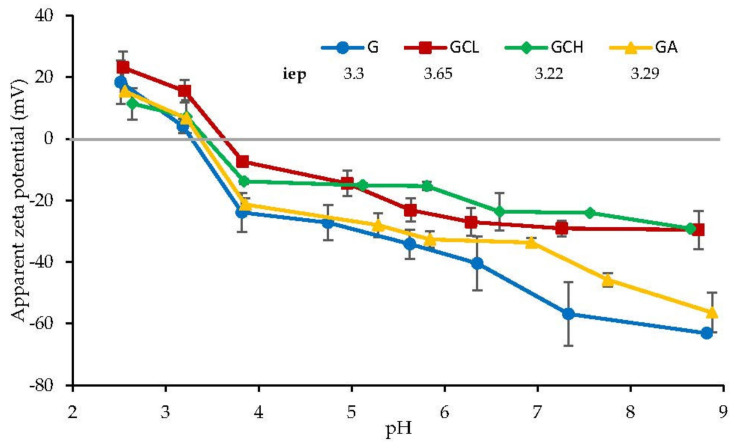
Zeta potential vs. pH profiles of the scaffolds (mean values ± sd; *n* = 3). In the inset, the isoelectric point (iep) is reported.

Figure 5 shows the ζ profiles vs. pH for the scaffolds. The isoelectric points (iep) of the scaffolds are given in the inset. All the scaffolds were characterized by iep in the range from pH 3 to 3.6. At pH lower than 3–3.6, the scaffolds showed a positive ζ, while a negative surface ζ was present in all the scaffolds when pH was higher than 3–3.6. This means that scaffolds should be negatively charged in the periodontal pocket environment, which has a pH ranging from 5 to 7 [36].

However, scaffolds possess a different pH-dependent behavior. In particular, chitosan-based scaffolds, GCL and GCH, showed a plateau above pH 5.5 with ζ close to −30 mV and different iep values, 3.65 and 3.22, respectively. This suggests that there is a prevalence of positively charged amino groups in GCH probably due to the CH deacetylation degree higher than that of CL. Furthermore, G and GA scaffolds were characterized by the lower ζ, close to −33/−50 mV at pH 7, respectively, with a less pronounced plateau.

It was recently reported that high surface potential enhances the collagen mineralization while the negative zeta potential in the physiological conditions (pH 7.4), seems to promote calcium mineralization, indispensable for tissue formation in the regeneration process in bone [37]. On the contrary, fibroblasts seem to better adhere and proliferate when the surface zeta potential is close to zero [38]. Moreover, bacterial adhesion was small on hydrophilic substrates with negative surface charge characteristics [39]. Considering all these features, the scaffolds possessed suitable surface properties to enhance cell adhesion and proliferation and to inhibit bacteria attachment.

### 3.3. Mechanical Properties

**Figure 6:**
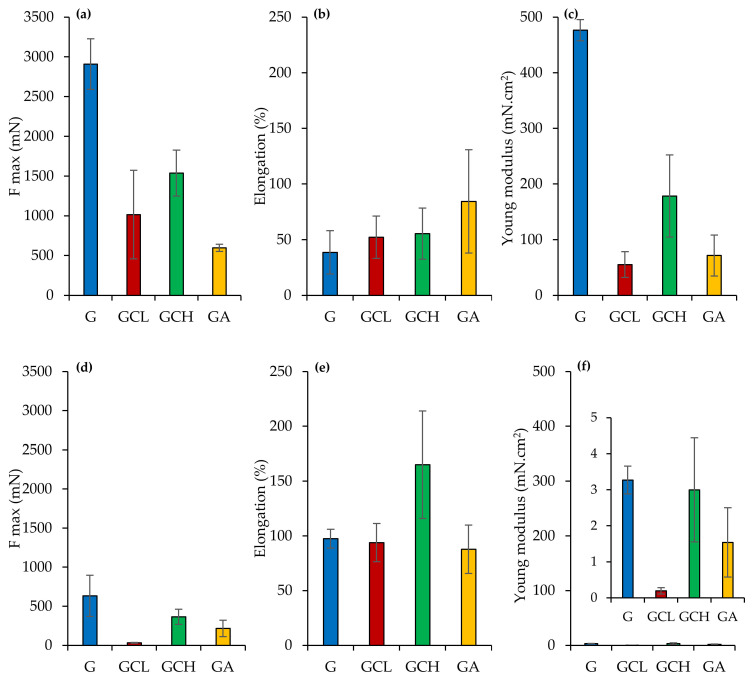
Mechanical properties (force at break mN, **a**–**d**; elongation %, **b**–**e**; Young’s modulus mN·cm^2^, **c**–**f**) for dry (**a**–**c**) and wet (**d**–**f**) scaffolds (mean values ± sd; *n* = 3). ANOVA one-way; Scheffé’s test (*p* < 0.05): (**a**) G vs. GCL, GCH, GA; GGH vs. GA; (**c**) = G vs. GCL, GCH, GA; GCL vs. GCH; GCH vs. GA; (**d**) = G vs. GCL, GA; GCL vs. GCH; (**a**) vs. (**d**) = G, GCL, GCH; (**b**) vs. (**e**) = GCH; (**c**) vs. (**f**) G, GCL, GCH, GA.

Figure 6 reports the mechanical properties (force at break mN, a,d; elongation %, b,e; Young’s Modulus mN cm^2^, c,f) of scaffolds in a dry (a,b,c) or wet (d,e,f) state.

In a dry state, G scaffold showed higher force at break and elasticity (Young’s modulus) combined with lower deformability, while the other scaffolds were characterized by significantly lower ones, although they presented deformability almost superimposable to G scaffolds. The presence of polysaccharides in the scaffolds seems to weaken the scaffold structure. Moreover, the hydration caused a significant decrease in both force at break and elasticity, while it increased the deformability, despite this being significantly different only for the GCH scaffold.

The superior mechanical properties of G scaffold could be related to the transition of random coil to α-helix conformation during the electrospinning process: acetic acid evaporation and the temperature decrease from 40 °C to 25 °C could lead to this phenomenon [40]. The mechanical properties in the dry state support the retention of scaffold integrity upon application in the periodontal pocket. Once placed, scaffold hydration occurs and although the mechanical properties decrease, the stress to be faced is minimal [27,41]. Although a compressive test could evaluate scaffolds mechanical properties when applied to bone, the tensile or flexural tests should point out scaffold resilience upon the implant into the periodontal pocket [42,43,44,45]. In particular, force at break, elongation and Young’s modulus should give information on flexibility and stiffness of the scaffolds, as an indicator to the system integrity in the periodontal pocket for supporting cell attachment and for enhancing cell proliferation to restore periodontal ligament.

### 3.4. In Vitro Cell Adhesion and Proliferation Assay

**Figure 7:**
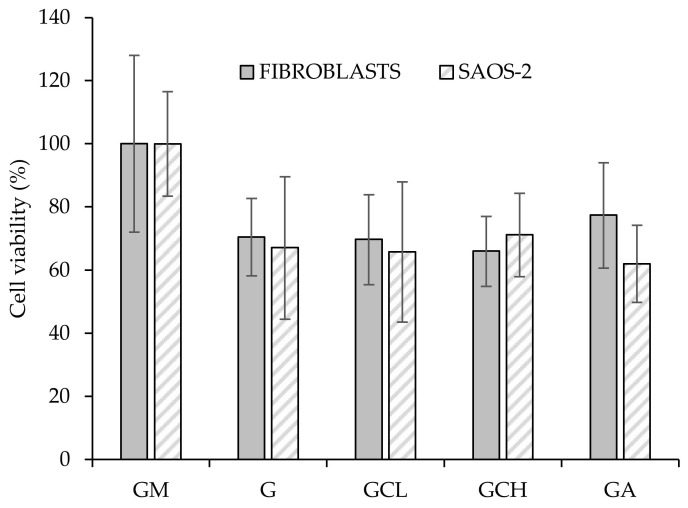
Cytocompatibility of fibroblasts and human primary osteogenic sarcoma cells (SAOS-2) grown for 6 days onto the scaffolds (mean values ± sd; *n* = 5). Abbreviation: GM growth medium.

Figure 7 reports the viability (%) of fibroblasts and SAOS-2 cells grown onto the scaffolds and in standard conditions (growth medium, GM) for 6 days.

All the scaffolds were able to support cell growth Independently of the cell type, and no significant differences between GM and scaffolds were observed.

**Figure 8:**
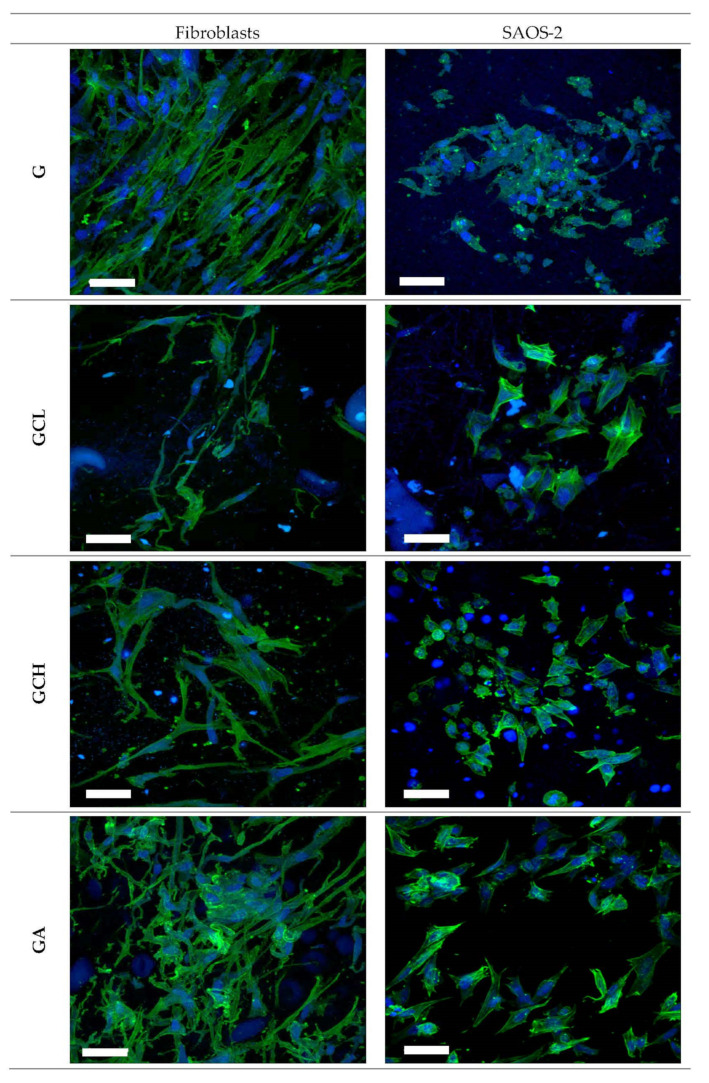
Confocal laser scanning microscopy (CLSM) (scale bar: 50 µm) images of fibroblasts and SAOS-2 grown for 6 days onto the scaffolds (in blue: nuclei; in green: cytoskeleton).

Moreover, CLSM analysis confirms scaffold biocompatibility (Figure 8). Fibroblasts and SAOS-2 cells grown onto the scaffolds maintained their normal morphology, characterized by fusiform and polygonal shapes, respectively. Adhesion and proliferation properties of both fibroblasts and osteoblasts are extremely important especially in severe periodontitis where there is loss of both gingival tissue and alveolar bone.

### 3.5. Microbiological Investigation

*Streptococcus mutans*, a Gram+ oral pathogen, was considered since it is known as the principal etiological agent in human dental caries. It is able to form biofilms on tooth surfaces causing dental plaque [46,47,48]. Furthermore, *Aggregatibacter actinomycetemcomitans* a Gram-, facultatively anaerobic was selected since it has a role in the most forms of periodontitis and in numerous oral infections [49,50].

The inhibition zone investigation suggests that the scaffold structure probably impaired the effectiveness of both high molecular weight chitosan (CH) and alginate (A) while the scaffold containing low molecular weight chitosan (CL) was effective against both the strains considered (diameters of the inhibition zones: *Streptococcus mutans*: 10.5 ± 0.5 mm; *Aggregatibacter actinomycetemcomitans*: 10.0 ± 1.0 mm). This was characterized by an effectiveness comparable to the reference, a chlorhexidine digluconate gel (1% *w*/*w*) (inhibition zone against *Streptococcus mutans* 13.0 ± 0.15 mm), a marketed dental gel commonly used locally to treat periodontitis.

There are many pieces of evidence in the literature related to the molecular weight of chitosan and its antimicrobial properties: in particular the lower the molecular weight the higher the antibacterial effect [51,52].

## 4. Conclusions

Nanofibrous scaffolds based on pristine gelatin and gelatin associated either chitosan (a low or a high molecular weight) or alginate were manufactured using electrospinning without the employment of toxic solvent (water and acetic acid mixture was used) and cross-liked by heating avoiding chemicals. The scaffolds were completely based on biopolymers and were intended as implants in the periodontal pocket to treat periodontitis.

The combination of gelatin with other biopolymers significantly affected the critical attributes of the scaffolds. However, the chemico–physical characterization suggests that all the scaffolds were found to be suitable for the intended use. Although the biocompatibility investigation evidences that all the scaffolds were able to support fibroblast and osteoblast adhesion and proliferation, the antimicrobial properties highlight that the scaffold based on gelatin and low molecular weight chitosan (GCL) was effective against both *Streptococcus mutans* and *Aggregatibacter actinomycetemcomitans*. Considering the suitable surface properties for enhancing cell adhesion and proliferation and for inhibiting bacteria attachment and the antimicrobial properties, the GCL scaffold seems a promising candidate for the treatment of periodontitis, particularly when gum and bone loss are also involved.

## Figures and Tables

**Table 1:** Quali-quantitative composition of the polymeric blends. Abbreviations: CH (high molecular weight chitosan), CL (low molecular weight chitosan), A (alginic acid sodium salt), G (gelatin), GCL (gelatin-low molecular weight chitosan), GCH (gelatin-high molecular weight chitosan) and GA (gelatin- alginic acid sodium salt)

% *w*/*w* (Blend Composition)	Gelatin	CH	CL	A	Citric Acid
G	20				5
GCH	20	2			5
GCL	20		2		5
GA	20			2	5

**Table 2:** Conductivity, pH, surface tension and viscosity values at 1000 s^−1^ of the blends (mean values ± sd; *n* = 3).

Formulation	Conductivity (µS/cm)	pH	Surface Tension (mN/m)	Viscosity (mPa·s)
G	3729 ± 611	3.6 ± 0.1	40.45 ± 0.5	131.1 ± 12.6
GCL	5186 ± 312	3.1 ± 0.1	38.14 ± 3.2	622.4 ± 144.0
GCH	3934 ± 518	3.0 ± 0.1	38.07 ± 3.3	678.5 ± 14.5
GA	4947 ± 103	2.9 ± 0.1	36.05 ± 0.1	390.1 ± 11.9

## Data Availability

Data available on request.

## References

[1] Genco R.J., Borgnakke W.S. (2013). Risk factors for periodontal disease. Periodontology.

[2] Tonetti M.S., Jepsen S., Jin L., Otomo-Corgel J. (2017). Impact of the global burden of periodontal diseases on health, nutrition and wellbeing of mankind: A call for global action. J. Clin. Periodontol..

[3] Chapple I.L.C. (2014). Time to take periodontitis seriously. BMJ.

[4] Quirybeb M., Bollen C.M., Vandekerckove B.N., Dekeyser C., Papaioannou W., Eyssen H. (1995). Full- vs. partial-mouth disinfection in the treatment of periodontal infections: Short-term clinical and microbiological observations. J. Dent. Res..

[5] Haffajjee A.D., Cugini M.A., Dibart S., Smith C., Kent R.L., Socransky S.S. (1997). The effect of SRP on the clinical and microbiological parameters of periodontal diseases. J. Clin. Periodontol..

[6] Drisko C.H. (2001). Nonsurgical periodontal therapy. Periodontol.

[7] American Academy of Periodontology (2000). Position paper: The role of controlled drug delivery for periodontitis. J. Periodontol..

[8] Zupancic S., Kocbek P., Baumgartner S., Kristl J. (2015). Contribution of nanotechnology to improved treatment of periodontal disease. Curr. Pharm. Des..

[9] Rajeshwari H.R., Dhamecha D., Jagwani S., Rao M., Jadhav K., Shaikh S., Puzhankara L., Jalalpure S. (2019). Local drug delivery systems in the management of periodontitis: A scientific review. J. Control. Release.

[10] Bapat R.A., Dharmadhikari S., Chaubal T.V., Amin M.C.I.M., Bapat P., Gorain B., Choudhury H., Vincent C., Kesharwani P. (2019). The potential of dendrimer in delivery of therapeutics for dentistry. Heliyon.

[11] Zamani M., Prabhakaran M.P., Ramakrishna S. (2013). Advances in drug delivery via electrospun and electrosprayed nanomaterials. Int. J. Nanomed..

[12] Asmatulu R., Khan W.S. (2019). Introduction to electrospun nanofibers. Synth. Appl. Electrospun. Nanofibers.

[13] Chou S.F., Carson D., Woodrow K.A. (2015). Current strategies for sustaining drug release from electrospun nanofibers. J. Control. Release.

[14] Chou S.F., Woodrow K.A. (2017). Relationships between mechanical properties and drug release from electrospun fibers of PCL and PLGA blends. J. Mech. Behav. Biomed. Mater.

[15] Monteiro A.P., Rocha C.M., Oliveira M.F., Gontijo S.M., Agudelo R.R., Sinisterra R.D., Segura M.E.C. (2017). Nanofibers containing tetracycline/β-cyclodextrin: Physico-chemical characterization and antimicrobial evaluation. Carbohydr. Polym..

[16] Zamani M., Morshed J., Varshosaz M., Jannesari M. (2010). Controlled release of metronidazole benzoate from poly e-caprolactone electrospun nanofibers for periodontal diseases. Eur. J. Pharm. Biopharm..

[17] Reise M., Wyrwa R., Müller U., Zylinski M., Völpel A., Schnabelrauch M., Berg A., Jandt K.D., Watts D.C., Sigusch B.W. (2011). Release of metronidazole from electrospun poly(l-lactide-co-d/l-lactide) fibers for local periodontitis treatment. Dent. Mater.

[18] Schkarpetkin D., Reise M., Wyrwa R., Völpel A., Berg A., Schweder M., Schnabelrauch M., Watts D.C., Sigusch B.W. (2016). Development of novel electrospun dual-drug fiber mats loaded with a combination of ampicillin and metronidazole. Dent. Mater.

[19] Budai-Szűcs M., Léber A., Cui L., Józó M., Vályi P., Burián K., Kirschweng B., Csányi E., Pukánszky B. (2020). Electrospun PLA fibers containing metronidazole for periodontal disease. Drug Des. Devel. Ther..

[20] Khan G., Yadav S.K., Patel R.R., Kumar N., Bansal M., Mishra B. (2017). Tinidazole functionalized homogeneous electrospun chitosan/poly (ε-caprolactone) hybrid nanofiber membrane: Development, optimization and its clinical implications. Int. J. Biol. Macromol..

[21] Kopytynska-Kasperczyk A., Dobrzynski P., Pastusiak M., Jarzabek B., Prochwicz W. (2015). Local delivery system of doxycycline hyclate based on -caprolactone copolymers for periodontitis treatment. Int. J. Pharm..

[22] Ranjbar-Mohammadi M., Zamani M., Prabhakaran M.P., Bahrami S.H., Ramakrishna S. (2016). Electrospinning of PLGA/gum tragacanth nanofibers containing tetracycline hydrochloride for periodontal regeneration. Mater Sci. Eng. C.

[23] Zheng F., Wang S., Wen S., Shen M., Zhu M., Shi X. (2013). Characterization and antibacterial activity of amoxicillin-loaded electrospun nano-hydroxyapatite/poly(lactic-co-glycolic acid) composite nanofibers. Biomaterials.

[24] Jain N., Jain G.K., Javed S., Iqbal Z., Talegaonkar S., Ahmad F.J., Khar R.K. (2008). Recent approaches for the treatment of periodontitis. Drug Discov. Today.

[25] Saporito F., Sandri G., Bonferoni M.C., Rossi S., Malavasi L., Del Fante C., Vigani B., Black L., Ferrari F. (2018). Electrospun gelatin—Chondroitin sulfate scaffolds loaded with platelet lysate promote immature cardiomyocyte proliferation. Polymers.

[26] Wiegand C., Heinze T., Hipler U. (2009). Comparative in vitro study on cytotoxicity, antimicrobial activity, and binding capacity for pathophysiological factors in chronic wounds of alginate and silver-containing alginate. Wound Repair Regen..

[27] Faccendini A., Ruggeri M., Miele D., Rossi S., Bonferoni M.C., Aguzzi C., Grisoli P., Viseras C., Vigani B., Sandri G. (2020). Norfloxacin-loaded electrospun scaffolds: Montmorillonite nanocomposite vs. free drug. Pharmaceutics.

[28] Sandri G., Faccendini A., Longo M., Ruggeri M., Rossi S., Bonferoni M.C., Miele D., Prina-Mello A., Aguzzi C., Viseras C. (2020). Halloysite- and montmorillonite-loaded scaffolds as enhancers of chronic wound healing. Pharmaceutics.

[29] Sandri G., Rossi S., Bonferoni M.C., Miele D., Faccendini A., Del Favero E., Di Cola E., Icaro Cornaglia A., Boselli C., Luxbacher T. (2019). Chitosan/glycosaminoglycan scaffolds for skin reparation. Carbohydrate Polymers.

[30] Malgarim Cordenonsi L., Faccendini A., Rossi S., Bonferoni M.C., Malavasi L., Raffin R., Scherman Schapoval E.E., Del Fante C., Vigani B., Miele D. (2019). Platelet lysate loaded electrospun scaffolds: Effect of nanofiber types on wound healing. Eur. J. Pharm. Biopharm..

[31] Sandri G., Miele D., Faccendini A., Bonferoni M.C., Rossi S., Grisoli P., Taglietti A., Ruggeri M., Bruni G., Vigani B. (2019). Chitosan/glycosaminoglycan scaffolds: The role of silver nanoparticles to control microbial infections in wound healing. Polymers.

[32] Birshtein V.Y., Tul’chinskii V.M. (1982). A study of gelatin by IR spectroscopy. Chem. Nat. Compd..

[33] Brugnerotto J., Lizardi J., Goycoolea F.M., Argüelles-Monal W., Desbrières J., Rinaudo M. (2001). An infrared investigation in relation with chitin and chitosan characterization. Polymer.

[34] Eastoe J.E., Ward A.G., Courtis A. (1977). Chemical constitution of gelatin. The Science and Technology of Gelatin.

[35] Mukherjee I., Rosolen M. (2013). Thermal transitions of gelatin evaluated using DSC sample pans of various seal integrities. J. Therm. Anal. Calorim..

[36] Vidal-Romero G., Zambrano-Zaragoza M.L., Martínez-Acevedo L., Leyva-Gómez G., Mendoza-Elvira S.E., Quintanar-Guerrero D. (2019). Design and evaluation of pH-dependent nanosystems based on cellulose acetate phthalate, nanoparticles loaded with chlorhexidine for periodontal treatment. Pharmaceutics.

[37] Metwally S., Ferraris S., Spriano S., Krysiak Z.J., Kaniuk L., Marzec M.M., Kim S.K., Szewczyk P.K., Gruszczyński A.K., Wytrwal-Sarna A.K. (2020). Surface potential and roughness controlled cell adhesion and collagen formation in electrospun PCL fibers for bone regeneration. Mater. Des..

[38] Hsun-Yun C., Chih-Chieh H., Kang-Yi L., Wei-Lun K., Hua-Yang L., Yun-Wen Y., Jiun-Hao L., Yu-Ting K., Ding-Yuan K., Jing-Jong S. (2014). Effect of surface potential on NIH3T3 cell adhesion and proliferation. J. Phys. Chem. C.

[39] Oh J.K., Yegin Y., Yang F., Zhang M., Li J., Huang S., Verkhoturov S.V., Schweikert E.A., Perez-Lewis K., Scholar E.A. (2018). The influence of surface chemistry on the kinetics and thermodynamics of bacterial adhesion. Sci. Rep..

[40] Sajkiewicz P., Kołbuk D. (2014). Electrospinning of gelatin for tissue engineering—molecular conformation as one of the overlooked problems. J. Biomater. Sci. Polym. Ed..

[41] Ruggeri M., Bianchi E., Rossi S., Vigani B., Bonferoni M.C., Caramella C., Sandri G., Ferrari F. (2020). Nanotechnology-based medical devices for the treatment of chronic skin lesions: From research to the clinic. Pharmaceutics.

[42] Requicha J.F., Viegas C.A., Hede S., Leonor I.B., Reis R.L., Gomes M.E. (2016). Design and characterization of a biodegradable double-layer scaffold aimed at periodontal tissue-engineering applications. J. Tissue Eng. Regen. Med..

[43] Qasim S.B., Najeeb S., Delaine-Smith R.M., Rawlinson A., Ur Rehman I. (2017). Potential of electrospun chitosan fibers as a surface layer in functionally graded GTR membrane for periodontal regeneration. Dent. Mater.

[44] Qasim S.B., Delaine-Smith R.M., Fey T., Rawlinson A., Rehman I.U. (2015). Freeze gelated porous membranes for periodontal tissue regeneration. Acta Biomater..

[45] Inanç B., Arslan Y.E., Seker S., Elçin A.E., Elçin Y.M. (2009). Periodontal ligament cellular structures engineered with electrospun poly(DL-lactide-co-glycolide) nanofibrous membrane scaffolds. J. Biomed. Mater. Res. A.

[46] Kuramitsu H.K. (1993). Virulence factors of mutans streptococci: Role of molecular genetics. Crit. Rev. Oral Biol. Med..

[47] Yamashita Y., Bowen W.H., Burne R.A., Kuramitsu H.K. (1993). Role of the Streptococcus mutans gtf genes in caries induction in the specificpathogen-free rat model. Infect. Immun..

[48] Yoshida A., Kuramitsu H.K. (2002). Multiple Streptococcus mutans genes are involved in biofilm formation. Appl. Environ. Microbiol..

[49] Raja M. (2014). Aggregatibacter actinomycetemcomitans—A tooth killer?. J. Clin. Diagn. Res..

[50] Oettinger-Barak O., Dashper S.G., Catmull D.V., Adams G.G., Sela M.N., Machtei E.E., Reynolds E.C. (2013). Antibiotic susceptibility of Aggregatibacter actinomycetemcomitans JP2 in a biofilm. J. Oral Microbiol..

[51] Sahariah P., Másson M. (2017). Antimicrobial chitosan and chitosan derivatives: A review of the structure-activity relationship. Biomacromolecules.

[52] Hosseinnejad M., Jafari S.M. (2016). Evaluation of different factors affecting antimicrobial properties of chitosan. Int. J. Biol. Macromol..

